# Meta-Analysis of Mixed Sowing Effects on Forage Yield and Water Use Efficiency in China: Influencing Factors and Optimal Conditions

**DOI:** 10.3390/plants14091283

**Published:** 2025-04-23

**Authors:** Weiqiang Guo, Yuanbo Jiang, Minhua Yin, Yi Ling, Yanxia Kang, Guangping Qi, Yaya Duan, Yanlin Ma, Yushuo Liu, Gen Ling, Kaili Pan

**Affiliations:** College of Water Conservancy and Hydrpower Engineering, Gansu Agricultural University, Lanzhou 730070, China; 1073323120772@st.gsau.edu.cn (W.G.); 1073323010121@st.gsau.edu.cn (Y.J.); 18693199350@163.com (Y.L.); qigp@gsau.edu.cn (G.Q.); 1073323020376@st.gsau.edu.cn (Y.D.); mayl@gsau.edu.cn (Y.M.); 20212201025@st.gsau.edu.cn (Y.L.); 20212201026@st.gsau.edu.cn (G.L.); 20212201021@st.gsau.edu.cn (K.P.)

**Keywords:** mixed sowing, forage, yield, water use efficiency, meta-analysis

## Abstract

Mixed sowing of forage grass can reduce soil erosion, improving forage nutritional composition, enhancing grassland productivity, and increasing community stability. It addresses issues faced by sown pasture, including a lack of diversity in planting patterns, low resource utilization efficiency, and poor sustainability. However, the effects of mixed sowing on forage yield and water use efficiency (WUE) vary depending on regional environmental conditions, management practices, and temporal factors. Based on publicly available field experiment data, this study utilized meta-analysis to quantitatively examine the effects of mixed sowing on forage yield and WUE in China. Additionally, a random forest model was employed to analyze the main influencing factors. The results showed that, compared with monoculture, mixed sowing significantly improved forage yield and WUE, with average increases of 58.3% (confidence interval: 44.3–72.3%) and 32.0% (confidence interval: 19.2–44.8%), respectively. Regarding yield, the effect of mixed sowing was the most pronounced in Shaanxi. Optimal conditions included experiments conducted during 2006–2008, annual precipitation of 200–600 mm, soil pH of 4−5, average annual temperature of 10–15 °C, altitudes below 2000 m, alfalfa (*Medicago sativa*) and *Bromus inermis* as the forage combination, two species in the mixture, a legume-to-grass species ratio of 1:1, a total seeding rate of 40–50 kg·ha^−1^, and mixed sowing in the same row. For WUE, significant effects were observed in Gansu under the following conditions: experiments conducted during 2018–2020, annual precipitation of 400–600 mm, an average annual temperature of 5–10 °C, a soil pH of 8–9, altitudes of 1000–2000 m, oats (*Avena sativa*) and peas (*Pisum sativum*) as the forage combination, two species in the mixture, a legume-to-grass species ratio of 1:1, a total seeding rate of <50 kg·ha^−1^, and mixed sowing in alternate rows. The random forest model indicated that the effects of mixed sowing on forage yield were primarily influenced by annual precipitation, average annual temperature, and experimental region. In contrast, the effects on WUE were mainly determined by forage combination, species type, and the legume-to-grass species ratio. This study provides a reference for enhancing alfalfa productivity and achieving efficient water use.

## 1. Introduction

Grasslands play an irreplaceable role in material cycling and energy transformation within ecosystems [1]. Globally, grassland resources are abundant, accounting for approximately 36–64% of the total green plant resources. However, excessive grazing and improper management practices have led to varying degrees of desertification, alkalization, and degradation of natural grasslands, resulting in decreased productivity and reduced livestock carrying capacity [2]. In recent years, the rapid development of the livestock industry, driven by population growth and increasing living standards, has intensified the conflict between grassland supply and livestock demand [3]. Establishing sown pasture can alleviate pressure on natural grasslands, allowing them to recover, while optimizing spatial and temporal grassland utilization [4,5]. However, sown pasture development in China is still in progress, with a total estimated area of approximately 5.8 × 10^5^ ha^−1^, accounting for only about 5% of the total grassland area [6,7]. In contrast, developed countries initiated sown pasture development much earlier, achieving more mature systems. For example, sown pasture/semi-sown pasture cover 66% of grassland in New Zealand and 29% in the United States [8]. Currently, sown pasture development in China faces challenges such as a relatively homogeneous planting structure, extensive field management practices, and poor forage quality, leading to a high dependence on imported high-quality forage [9]. Furthermore, in the context of increasing global water scarcity, reducing the dependence of forage production on water resources has become particularly important [10]. Compared with monoculture, mixed sowing has been shown to improve soil quality, enhance resistance to erosion and stress, and increase water use efficiency (WUE) and forage quality. It is considered a key strategy to mitigate the conflict between grassland and livestock development while reducing pressure on water resources and the ecological environment [11,12].

Compared with monoculture, mixed sowing enables a more efficient utilization of soil nutrients, improves soil structure, reduces weed and disease pressure, and mitigates water and soil loss. These benefits collectively enhance forage nutritional composition and grassland productivity, providing significant economic, social, and ecological advantages. As a result, mixed sowing has become a primary mode of sown pasture establishment [13]. A well-planned forage combination and appropriate mixing ratio can maximize spatial, temporal, and energy-use complementarity within the plant community, thereby enhancing interspecies interactions and improving yield, stress resistance, and community stability [14]. Artificial mixed grasslands are primarily composed of mixtures of Leguminosae and Gramineae, commonly referred to as legume–grass mixed sowing (LGM). Studies have shown that in LGM systems, the fibrous root system of Gramineae complements the taproot system of Leguminosae, effectively adjusting interspecies competition and mitigating environmental constraints [15]. In legume–grass mixed sowing, Leguminosae contributes nitrogen fixation by forming symbiotic relationships with rhizobia, thereby providing nitrogen to its companion Gramineae and reducing nitrogen fertilizer requirements [16]. Meanwhile, the extensive root systems of Gramineae help loosen the soil and promote legume growth, creating a mutually beneficial system that enhances soil fertility [17]. Furthermore, legume forages are rich in protein, while excessive consumption of Leguminosae alone can cause bloating in livestock. Conversely, Gramineae has a lower protein content. Mixing Leguminosae with Gramineae balances livestock nutrition and prevents digestive disorders [18]. For instance, in alfalfa–grass mixed sowing systems, alfalfa maintains green foliage longer in autumn, extending the grazing period and optimizing grassland utilization [19]. Mixed sowing also maximizes the use of sunlight, heat, and water resources, enhancing photosynthetic efficiency and increasing dry matter accumulation, thereby improving forage yield [20]. Studies have found that white clover–alfalfa mixed sowing benefits from white clover’s stolon, which cover the ground and reduce water evaporation, while alfalfa’s deep roots access nutrients and moisture from deeper soil layers. This complementary effect results in higher total yield and improved water use efficiency [21]. Different forage species possess distinct nutritional compositions, and mixed sowing allows for complementary nutrient profiles, thereby improving forage quality [22]. For example, studies have shown that oat–barley mixed sowing enhances feed value, since oats are rich in protein and fat, while barley provides more carbohydrates, making the mixture more palatable and nutritionally balanced [23]. Despite its advantages, the effects of mixed sowing on forage production vary significantly across different studies. This variation indicates that the effectiveness of mixed sowing is influenced by environmental conditions, soil characteristics, and management practices. If incompatible forage species are mixed, competition for resources such as light, water, and nutrients may intensify, leading to reduced community stability and productivity [24,25]. For example, studies have found that excessive height differences between forage species can result in shading, where taller species block sunlight from reaching shorter ones, thereby reducing their photosynthetic capacity and limiting growth [26]. Similarly, taller species with extensive root systems may outcompete shorter species for water and nutrients, further suppressing their growth. In arid regions, mixing drought-tolerant and non-drought-tolerant forage species may result in poor growth of the latter due to inadequate water availability, ultimately lowering grassland productivity [27]. Additionally, inappropriate mixed sowing strategies can lead to reduced forage yield and quality [28,29,30]. Research has demonstrated that in alfalfa–grass mixed sowing systems, the excessively rapid growth of Gramineae can result in single-species dominance, thereby reducing biodiversity and negatively impacting alfalfa yield and quality, ultimately lowering the overall value of the mixed grassland [31].

In conclusion, there are great differences in the production effect of forage mixed sowing between different studies. These results indicated that the effects of mixed sowing on forage production were influenced by environmental conditions, soil characteristics, planting management, and other factors. In order to give full play to the advantages of mixed seeding and maximize the production effect of mixed sowing herbage, this study selected yield and water use efficiency, the core indicators of the production performance of mixed sowing grassland, as the target, and adopted meta-analysis method to integrate the published literature on mixed seeding herbage yield and water use efficiency, aiming to achieve (1) a quantitative analysis of the effects of mixed sowing on forage yield and water use on a national scale; (2) a exploration of how the effects vary in regards to the different environmental factors and management practices; (3) the provision of a theoretical basis and technical support for promoting the planting level of sown pasture and the efficient and sustainable development of forage.

## 2. Results and Analysis

### 2.1. Sample Distribution of Forage Yield and Water Use Efficiency

**Figure 1:**
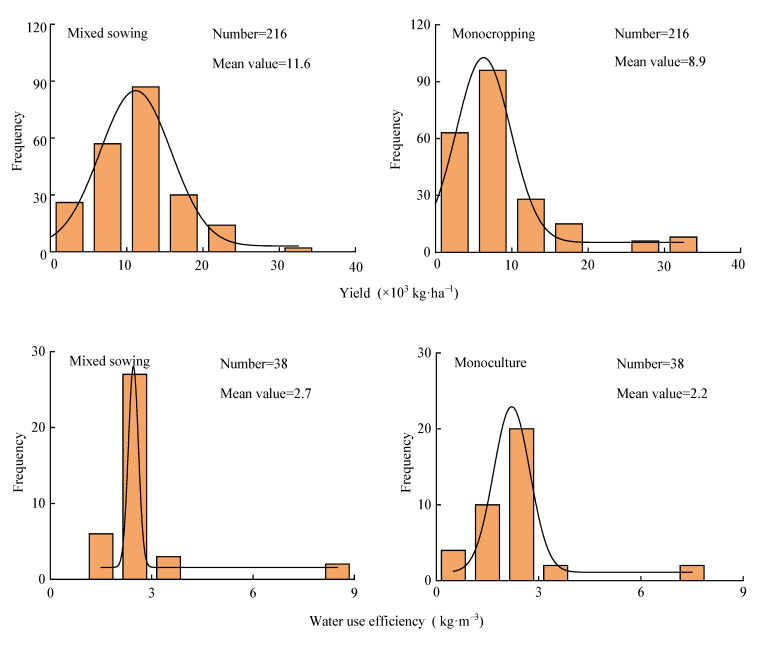
Sample distribution of forage yield and water use efficiency under mixed sowing and monoculture conditions.

Under mixed sowing and monoculture conditions, the samples of forage yield and water use efficiency (WUE) were approximately normally distributed (Figure 1). The forage yield and WUE under mixed sowing ranged from 301.7 to 31,200 kg·ha^−1^ (average 11,608.3 kg·ha^−1^) and 1.4 to 8.3 kg·m^−3^ (average 2.7 kg·m^−3^), respectively. In comparison, the forage yield and WUE under monoculture ranged from 100 to 32,424 kg·ha^−1^ (average 8942.1 kg·ha^−1^) and 1.1 to 9.8 kg·m^−3^ (average 2.2 kg·m^−3^), respectively.

### 2.2. Overall Effects of Mixed Sowing on Forage Yield and Water Use Efficiency

The calculation of the overall effect size (Table 1) revealed that the heterogeneity test results for forage yield and water use efficiency under mixed sowing were both statistically significant (*P_Q_* < 0.001); thus a random-effects model was applied. The random forest interpretation of yield was 70%, and that of WUE was 60.0%. Overall, compared with monoculture, mixed sowing significantly increased forage yield and water use efficiency by an average of 58 ± 13% and 32 ± 14%, respectively.

### 2.3. Factors Influencing Forage Yield Under Mixed Sowing

#### 2.3.1. Regional Factors

**Figure 2:**
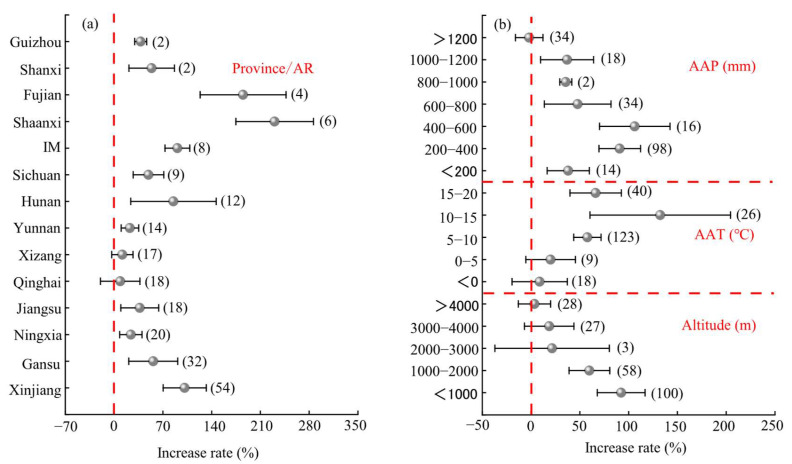
Analysis of factors influencing forage yield under mixed sowing. (**a**) Regional factors, (**b**) precipitation, temperature and altitude factors AR: autonomous region; AAP: average annual precipitation; AAT: average annual temperature.

Regional factors, including province/autonomous region, annual precipitation, annual average temperature, and altitude, significantly influenced the yield effects of mixed sowing (Figure 2a). Among the provinces and regions, the highest yield increase effect of mixed sowing was observed in Shaanxi (average: 231 ± 64%), but this was not significantly different from that in Fujian (average: 185 ± 70%). The effects for Shanxi (average: 54.0%), Inner Mongolia (average: 90.9%), Sichuan (average: 49.4%), Hunan (average: 85.2%), Gansu (average: 56.3%), and Xinjiang (average: 101.4%), were exhibited varying results, while those for Guizhou (average: 38.1%), Yunnan (average: 22.7%), Jiangsu (average: 36.9%), and Ningxia (average: 24.1%) showed lower yield effects. The lowest yield increase effect was observed in Qinghai (average: 8.8%). Regarding climatic factors (Figure 2b), the yield effect of mixed sowing increased initially and then decreased with rising annual precipitation and average annual temperature. The highest yield effect was observed when the annual precipitation was 400–600 mm (average: 106 ± 41%), although this was not significantly different from the 200–400 mm range. Similarly, the highest yield effect occurred at an average annual temperature of 10–15 °C (average: 132 ± 71%), with no significant difference compared to the effects at 5–10 °C and 15–20 °C. Additionally, the yield effect of mixed sowing decreased with increasing altitude. The highest yield effect was observed at altitudes below 1000 m (average: 92 ± 31%), but it was not significantly different from the effects at altitudes of 1000–2000 m (average: 59.8%). At altitudes above 2000 m, the yield effect was not significant.

#### 2.3.2. Mixed Sowing Factors

**Figure 3:**
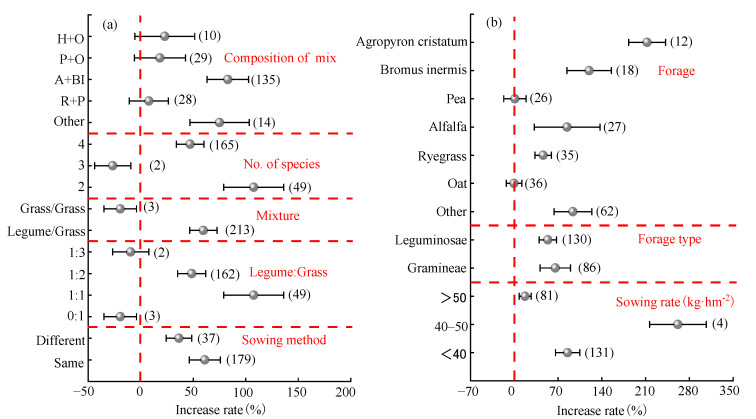
Analysis of factors influencing forage yield under mixed sowing (**a**) and monoculture (**b**). H + O: hairy vetch + oats; P + O: peas + oats; A + BI: alfalfa + Bromus inermis; R + P: ryegrass + peas.

Mixed sowing factors primarily include composition of mix, no. of species, mixture, legume–grass ratio, and sowing method, (Figure 3a). The yield increase effect of mixed sowing was highest when alfalfa + *Bromus inermis* were combined (average: 83 ± 21%), whereas the yield effects of hairy vetch + oats (average: 23.0%), peas + oats (average: 18.4%), and ryegrass + peas (average: 7.8%) were not significant. When the number of forage allocations was two, the yield increase effect of mixed sowing was significantly the highest (average: 108 ± 37%). The effect was lower with four allocations (average: 47 ± 18%) and significantly decreased by 26.5% with three allocations. Mixed sowing of Leguminosae and Gramineae significantly increased the yield by 59.7% (95% CI: 42.6–76.8%), while mixing Gramineae with Gramineae resulted in a significant yield decrease of 19.4%. The yield increase rate of mixed sowing decreased as the proportion of Gramineae in the legume-to-grass ratio increased. The maximum yield increase effect was observed when the species ratio of Leguminosae to Gramineae was 1:1 (average: 108 ± 28%). Mixed sowing in the same rows exhibited a higher yield increase effect (average: 61 ± 16%), although the difference from mixed sowing in difference rows (average: 37 ± 13%) was not significant.

#### 2.3.3. Monoculture Factors

Forage, forage type, and sowing rate significantly influenced forage yield (Figure 3b). Among forage species, the highest yield increase effect was observed for *Agropyron cristatum* (average: 213 ± 27%), followed by *Bromus inermis* (average: 119.8%), alfalfa (average: 84.6%), and other forage species (average: 93.7%). Ryegrass showed the lowest yield increase effect (average: 46.3%), while the yield effects of peas (average: 1.1%) and oats (average: −0.3%) were not significant

#### 2.3.4. Other Factors

**Figure 4:**
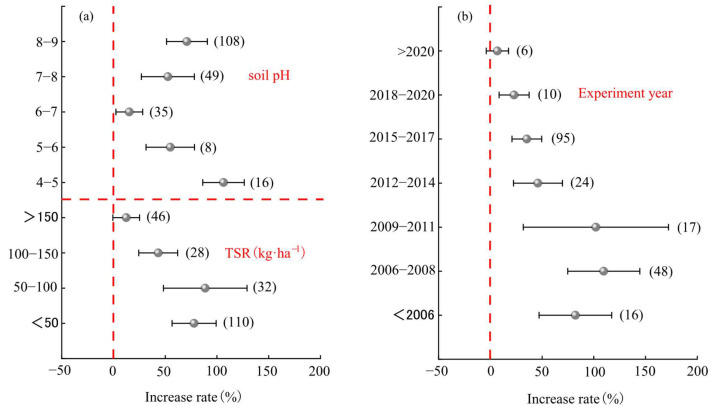
Analysis of the influence of other factors on forage yield. (**a**) Soil pH and total seeding rate factors; (**b**) Experiment year factors.

Soil pH, total sowing rate (Figure 4a), and experimental year (Figure 4b) significantly influenced the yield of mixed forage. With increasing soil pH, the yield effect of mixed sowing initially decreased and then increased. The highest average yield increase effect was observed at a soil pH of 4–5 (average: 106 ± 20%), although it was not significantly different from that at a soil pH of 8–9 (average: 71.0%). The yield increase effect of mixed forage initially rose and then declined with increasing total sowing rate and advancing experimental years. The highest average yield increase effects were observed at a total sowing rate of 50–100 kg·ha^−1^ (average: 89 ± 41%) and during the experimental years 2006–2008 (average: 110 ± 38%).

### 2.4. Factors Influencing Water Use Efficiency of Mixed Sowing

#### 2.4.1. Regional Factors

**Figure 5:**
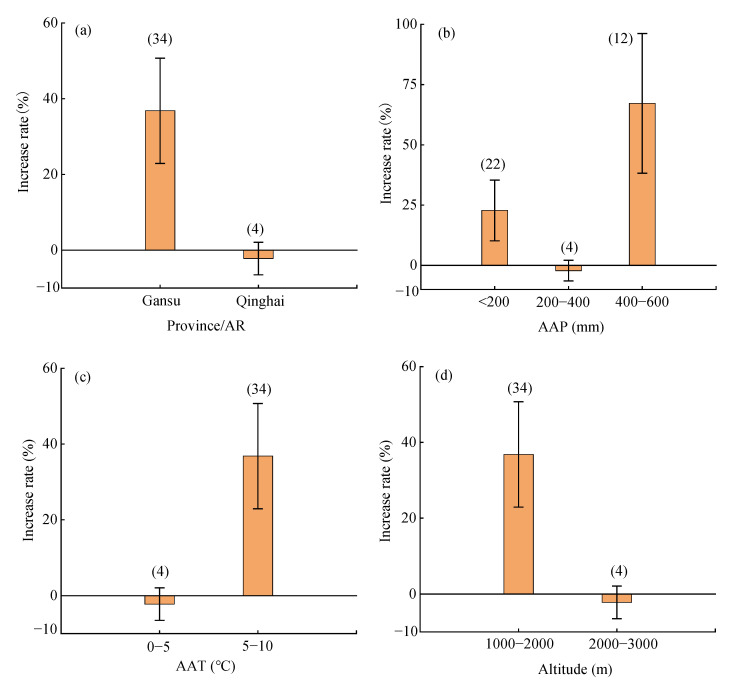
Effects of regional factors on water use efficiency of mixed forage sowing. (**a**) Province/AR factors (**b**) average annual precipitation factors (**c**) average annual temperature factors (**d**) Altitude factors AR: autonomous region; AAP: average annual precipitation; AAT: average annual temperature.

Region, average annual precipitation, average annual temperature, and altitude significantly influenced the water use efficiency (WUE) of mixed forage (Figure 5). In Gansu, the average WUE of mixed forage increased significantly by 36.8% (95%CI: 22.8–52.3%, Figure 5a), while the effect in Qinghai was not significant. As the average annual precipitation increased, the effect of mixed sowing on WUE showed a decreasing-then-increasing trend (Figure 5b). The highest increase in WUE was observed at an annual precipitation of 400–600 mm (average: 67 ± 32%). At an average annual temperature of 0–5 °C, WUE showed an insignificant decrease of −7.1–2.7%, while at 5–10 °C, it increased significantly by 22.8–52.3% (Figure 5c). At altitudes of 1000–2000 m and 2000–3000 m, WUE showed a significant increase of (22.8–52.3%) and an insignificant decrease of −7.1–2.7%, respectively (Figure 5d).

#### 2.4.2. Mixed Sowing Factors

**Figure 6:**
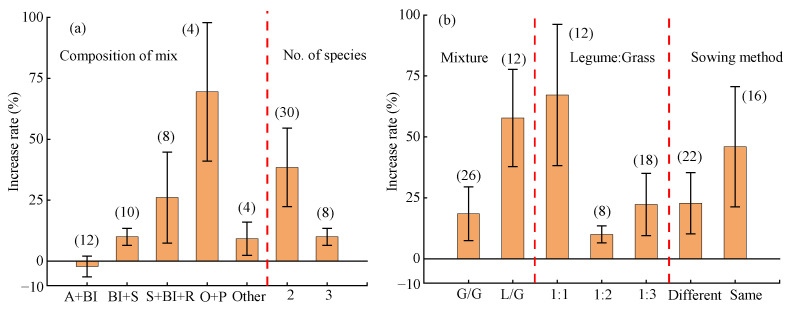
Effects of mixed sowing factors on water use efficiency of mixed forage. (**a**) composition of the mixture and No. of species factors (**b**) mixture and legume:grass and sowing method factors. A + BI: alfalfa + *Bromus inermis*; BI + S: *Bromus inermis* + sainfoin; S + BI + R: sainfoin + *Bromus inermis* + ryegrass; O + P: oats + peas.

Composition of mix, no. of species, (Figure 6a), mixture, legume–grass ratio, and sowing method (Figure 6b) significantly influenced the water use efficiency (WUE) of mixed forage sowing. The highest increase in WUE was observed with the combination of oats + peas (average: 70 ± 32%), although this was not significantly different from that for sainfoin + *Bromus inermis* + ryegrass (average: 26 ± 16%). The average WUE increase effect was significantly higher when the number of forage allocation was two (average: 39 ± 18%) compared to three (Average: 10 ± 3%). Mixtures of Leguminosae and Gramineae resulted in a significantly higher WUE increase effect (average: 58 ± 22%) compared to that for Gramineae only (average: 19 ± 12%). When the species ratio of Leguminosae to Gramineae was 1:1, the WUE increase effect of mixed sowing was the highest (average: 67 ± 32%), and was significantly greater than that for ratios of 1:3 and 1:2. Compared to mixed sowing in same rows (average: 23 ± 14%), mixed sowing in different rows (average: 46 ± 28%) showed a higher WUE increase effect, although the difference was not significant.

#### 2.4.3. Monoculture Factors

**Figure 7:**
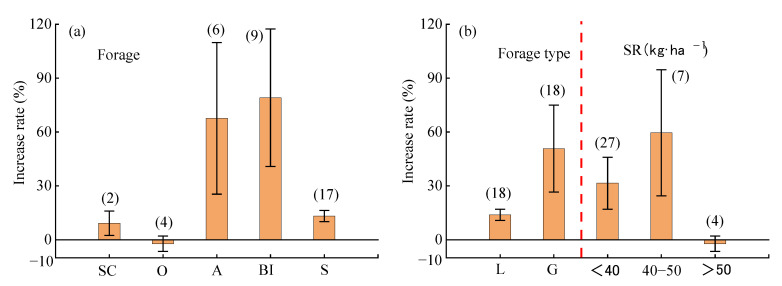
Effects of monoculture factors on water use efficiency of mixed forage. (**a**) Forage factor (**b**) Forage type and seeding rate. SC: silage corn; O: oats; A: alfalfa; BI: *Bromus inermis*; S: sainfoin; L: Leguminosae; G: Gramineae; SR: sowing rate.

Forage (Figure 7a), forage type, and monoculture sowing rate (Figure 7b) significantly influenced WUE. The highest WUE increase effect was observed with *Bromus inermis* monoculture (average: 73 ± 39%), but this was not significantly different from the results for alfalfa monoculture (average: 68 ± 42%). Sainfoin monoculture and silage corn monoculture followed, while oat monoculture (average: −2.2%) showed no significant effect on WUE. The WUE increase effect of monoculture was significantly higher for Gramineae (average: 51 ± 22%) compared to Leguminosae (average: 14 ± 2%). With increasing sowing rates, the WUE increase effect initially rose and then declined, with the highest effect observed at a sowing rate of 40–50 kg·ha^−1^ (average: 60 ± 40%).

#### 2.4.4. Other Factors

**Figure 8:**
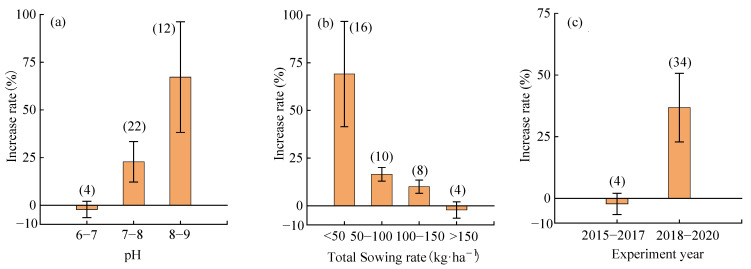
Effects of other factors on water use efficiency of mixed forage. (**a**) pH factor (**b**) total seeding rate factor (**c**) experiment year factor.

Soil pH (Figure 8a), total sowing rate (Figure 8b), and experimental year (Figure 8c) also significantly influenced the water use efficiency (WUE) of mixed forage sowing. The WUE increase effect of mixed forage sowing significantly improved with increasing soil pH, reaching its highest at a soil pH of 8–9 (average: 67 ± 32%). The WUE increase effect decreased with rising total seeding rates (Figure 8b), with the highest improvement observed at a total seeding rate of <50 kg·ha^−1^ (average: 69 ± 30%). For experimental years 2015–2017, the WUE showed an insignificant decrease (−7.1–2.7%), whereas for 2018–2020, it significantly improved (average: 22 ± 15%).

### 2.5. Importance of Mixed Sowing for Forage Yield and Water Use Efficiency

**Figure 9:**
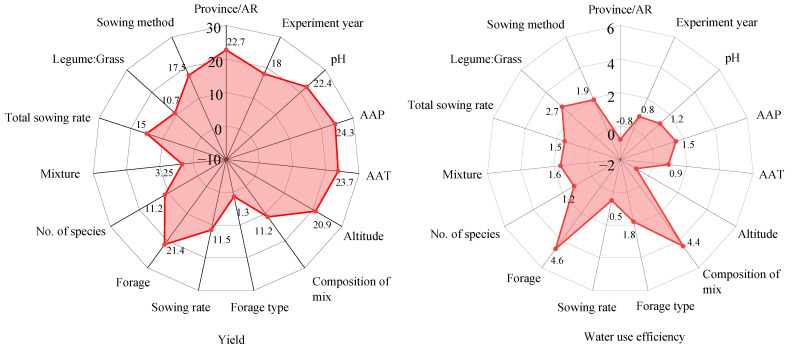
Analysis of the impact of mixed sowing on forage yield and water use efficiency. AR: autonomous region; AAP: average annual precipitation; AAT: average annual temperature.

The importance of factors influencing the yield and water use efficiency (WUE) improvement effects of mixed sowing was analyzed using a random forest model (Figure 9). For yield, among the top three influencing factors, the first comprises annual average precipitation (24.3%), annual average temp (23.7%), provinces/AR (22.7%), and soil Ph (22.4). The second is forage (21.4), and the third is altitude (20.9). The bottom three factors were the species ratio of Leguminosae to Gramineae (10.7%), forage allocation type (3.3%), and forage allocation (1.3%). For WUE, the top three influencing factors were forage allocation (4.6%), forage type (4.4%), and species ratio of Leguminosae to Gramineae (2.7%). The bottom three factors were experimental year (0.8%), experimental region (−0.8%), and altitude (−0.9%). It can be seen that regional factors (provinces/AR, annual average precipitation, average annual temperature, altitude, etc.) have a greater influence than management factors on mixed forage yield, whereas for mixed forage WUE, this effect is not significant due to the small sample size.

## 3. Discussion

### 3.1. Effects of Mixed Sowing on Forage Yield and Water Use Efficiency

From the perspectives of forage yield, quality, and production stability, mixed sowing is considered an optimal approach for cultivated grasslands [32]. The mixed sowing of forage species with different ecological niches allows for better adaptation to environmental conditions, enhances stress resistance, improves grassland ecological stability, and prolongs grassland lifespan [33]. By integrating existing studies on forage yield and water use efficiency in mixed sowing systems, this study found that, compared with monoculture, mixed sowing significantly increased forage yield and water use efficiency by 58.3% and 32.0%, respectively. Zhou et al. [34] reported that mixed sowing increased productivity by 14.0–43.2% compared to the results for monoculture, while Du et al. [35] found that mixed sowing resulted in a seven-fold increase in forage yield compared to the results for monoculture *Bromus inermis*. These findings collectively confirm that mixed sowing, as an efficient cultivation system, plays a significant role in increasing forage yield. The increased yield in mixed sowing systems may be attributed to the complementary resource use facilitated by species diversity. Differences in root distribution, nutrient requirements, and growth cycles among forage species allow mixed sowing systems to maximize the utilization of soil nutrients and water. Additionally, biological complementarity and symbiotic interactions among species can improve soil conditions, enhance stress resistance, and stabilize production, ultimately leading to higher forage yield and water use efficiency. However, Zhang et al. [36] reported that alfalfa–grass mixed sowing resulted in lower yields than alfalfa monoculture [37]. This may be due to competition between the two plant species in mixed systems. Alfalfa, having a strong competitive advantage due to its rapid growth and extensive root system, may suppress the growth of companion Gramineae, leading to reduced biomass accumulation in grass species.

### 3.2. Influence of Regional Factors on Forage Yield and Water Use Efficiency in Mixed Sowing

The production effects of mixed sowing vary across regions due to the combined influences of climate conditions, soil properties, topography, and human activities [38]. Moreover, the demand for commercial forage in China is concentrated in key dairy farming regions such as Inner Mongolia, Ningxia, Hebei, Tianjin, Shandong, Shanghai, Jiangsu, Beijing, Guangdong, Sichuan, and Chongqing. This has resulted in a “north-to-south” and “west-to-east” forage transportation pattern in China [39]. This study found that the yield increase effect of mixed sowing was the highest in Shaanxi and Fujian provinces. This may be because Shaanxi has a temperate continental monsoon climate with distinct seasons, abundant sunlight, and significant diurnal temperature variation, all of which promote photosynthesis and nutrient accumulation, leading to increased forage yield [40]. meanwhile, Fujian exhibits advanced agricultural techniques and adopts scientific planting and management practices, contributing to improved forage production levels. Unlike the findings of Zhou et al. [26] and Xie et al. [41] regarding forage mixed sowing, this study found that mixed sowing displayed no significant yield improvement effects in Qinghai and Tibet. This discrepancy may be related to differences in forage species composition and mixing ratios, It may also be due to different sample sizes. In terms of water use efficiency, mixed sowing exerted the highest improvement effect in Gansu, consistent with the findings of Yin et al. [3] in alfalfa studies. A possible reason is that Gansu is one of the most water-scarce provinces in China, and mixed sowing can effectively enhance root morphology and soil physicochemical properties, thereby improving water use efficiency. Climate factors regulate soil moisture dynamics and forage physiological processes [42,43], making them critical determinants of mixed sowing effectiveness. Insufficient precipitation can limit plant growth, while excessive precipitation may lead to waterlogging, affecting root respiration. Temperature also influences physiological processes such as photosynthesis and respiration, thereby affecting plant growth and yield. Different grass species display different adaptabilities to temperature, and extreme high or low temperature may affect the competitiveness of some grass species, resulting in changes in the proportion of mixed sowing. [44]. This study found that when annual precipitation ranged from 400 to 600 mm, mixed sowing had the greatest positive effect on forage yield and water use efficiency. When the average temperature was 10~15 °C and 5~10 °C, the yield and water use efficiency of mixed feed showed the greatest increase. Ehsan et al. [45] also reported similar findings regarding the effects of climate on crop yields. A likely explanation is that forage species generally prefer warm and moderately dry climates but also require adequate moisture. Excess soil moisture during the growing season may lead to waterlogging, disease outbreaks, and reduced productivity, whereas inadequate moisture can delay regrowth of the crop after the winter thaw, shortening the growing period and reducing the yield. Additionally, optimal precipitation levels can increase forage coverage, allowing mixed sowing systems to better utilize light, heat, and other resources, thereby enhancing photosynthesis and nutrient absorption efficiency. Altitude also influences temperature and precipitation, indirectly affecting forage growth. Different altitudes are suitable for different forage species [46,47]. High-altitude areas favor cold-tolerant species, whereas low-altitude areas are more suitable for heat-tolerant species. This study found that at altitudes of 1000–2000 m, mixed sowing exerted the highest improvement effect on forage yield and water use efficiency. This may be because soil fertility in this altitude range is relatively high, providing sufficient nutrients for forage growth. Additionally, soil moisture retention capacity at this altitude is stronger, effectively reducing evaporation and enhancing water use efficiency. However, at excessively high altitudes, increased solar radiation and inadequate accumulated temperature can lead to stunted plant growth and delayed development [48]. In this study, it is concluded that the optimal planting pattern proposed in this work can better improve yield and WUE in semi-arid, medium altitude locations, with an average annual temperature of about 10 °C. Thus, rational utilization of regional resources and optimized management practices can enhance the productivity and water use efficiency of mixed sowing systems. The sample distribution in this study displays a clear regional concentration, and 47.2% of the data comes from Gansu and Xinjiang provinces. This regional limitation may affect the applicability of the conclusions of the study to a wider geographical range, which needs to be further investigated in future trials.

### 3.3. Influence of Mixed Sowing and Monocultureg Factors on Forage Yield and Water Use Efficiency

The key to establishing artificial mixed grasslands lies in selecting appropriate species combinations and optimizing mixing ratios [49]. However, inappropriate combinations, poor management, and environmental constraints often lead to poor forage growth, intensified interspecies competition, reduced community stability, and weakened resilience to disturbances, ultimately affecting grassland productivity [50,51]. This study found that, compared with monoculture, a 1:1 legume-to-grass mixing ratio—particularly in alfalfa + *Bromus inermis* and oats + peas mixed systems—resulted in higher forage yield and water use efficiency. This aligns with the findings of Lan et al. [52], Ma et al. [53], and Zheng et al. [54]. In legume–grass mixed systems, the fibrous root systems of Gramineae facilitate efficient water and nutrient absorption, supporting the biological nitrogen fixation ability of Leguminosae under limited water conditions. This complementary effect helps balance nitrogen availability in grassland ecosystems, enhances soil fertility, and improves forage yield and quality. Furthermore, Leguminosae displays an open canopy structure that enhances light penetration, while Gramineae exhibits narrower leaves, with lower light transmission. Mixed sowing significantly improves light utilization efficiency [55]. Mixing ratios play a key role in the adaptability and stability of mixed grasslands. When the legume-to-grass ratio is 1:1, the total community density and species population density may be more favorable for forage growth. For example, in alfalfa + *Bromus inermis* mixed sowing, underground and aboveground spatial arrangements are more rational, and their differing growth cycles allow for better utilization of the growing season [22]. Oats + peas mixed sowing benefits from the high leaf density of oats, which provides shade and reduces direct sunlight exposure, aiding in pea seedling emergence and growth [56]. Additionally, the stress resistance and extensive root system of oats contribute to improved soil structure, enhanced aeration, and increased water retention, reducing evaporation and runoff, thereby providing peas with a better growth environment and nutrient supply. This study also found that compared with monoculture, mixed sowing involving two forage species resulted in greater improvements in forage yield and water use efficiency. This is consistent with the findings of Zhu et al. [57], likely because two-species mixtures form a balanced complementary relationship rather than a competitive one. However, when three species were mixed, forage yield significantly declined, although the sample size was small (only two cases), limiting its representativeness. Furthermore, both row-based and inter-row mixed sowing significantly improved yield and WUE compared to that of monoculture, with no significant difference between the two. This may be because row-based mixed sowing optimally utilizes space, increasing diversity and enhancing grassland productivity and stability, while inter-row mixed sowing enhances complementarity, improving overall yield and quality while reducing pest incidence.

### 3.4. Influence of Other Factors on Forage Yield and Water Use Efficiency

Sowing rate, soil pH, and experimental year significantly influence the yield and water use efficiency of mixed-sown forage. Sowing rate is a key parameter for regulating crop density and plays an important role in modulating the resource use efficiency and productivity of mixed-sown forage [58,59]. A scientifically rational sowing rate can effectively coordinate the relationship between individual plants and the population and fully exploit the potential of light, heat, and space, as well as water and fertilizer resources. This study shows that under different sowing rates, the yield and water use efficiency of mixed-sown forage are significantly increased. Among them, the highest yield increase rate of forage is achieved at a sowing rate of 50–100 kg·ha^−1^, while the highest rate of increase in water use efficiency is observed at a sowing rate of less than 50 kg·ha^−1^. This indicates that the effect of seeding rate on the yield and water use efficiency of mixed-sown forage has a certain threshold effect, thus requiring optimization based on specific objectives [60].

Soil pH serves as a critical indicator of soil salinization and alkalization, influencing the activity of microbial metabolic enzymes and thereby affecting nutrient absorption by forage. This study revealed that in soils with a pH between 4 and 5, the rate of yield increase for mixed sowing forages is relatively high; conversely, when the soil pH ranges from 8 to 9, water use efficiency in mixed sowing forages is enhanced. In acidic soils, the availability of nutrients such as phosphorus and potassium is elevated, facilitating their uptake and utilization by forage Gramineae, which promotes growth and development and consequently increases yield. In alkaline soils, higher carbonate content improves soil aeration and permeability [61], fostering root growth and water absorption in forage Gramineae. Additionally, sodium ions and other components in alkaline soils regulate transpiration in forage Gramineae, effectively enhancing their water use efficiency Acidic or alkaline soil may inhibit the growth of some grass species, affecting the stability of mixed sowing [62].

The experimental year also significantly affected the yield and water use efficiency of mixed-sown forage. With the progression of the experimental years, there was a significant interannual fluctuation in the production effect of mixed-sown forage. This study found that the yield increase rate of mixed sowing forage showed an upward trend before 2011, but began to decline after 2011. The policy document titled “Opinions of the Central Committee of the Communist Party of China and the State Council on Several Policies to Promote Farmers’ Income Increase”, issued in 2004, significantly increased investment in agricultural infrastructure and technological services. These measures improved agricultural production conditions and enhanced the comprehensive agricultural production capacity, thereby driving the increase in the yield of mixed-sown forage. However, From 2009 to 2011, the forage yield fluctuated greatly, and the implementation of the Grassland Ecological Protection Subsidy and Reward Mechanism in 2011 may have led to a significant decrease in forage yield. In addition, factors such as variety degeneration, declining soil fertility, and increased environmental stress may also have contributed to the decline in the yield of mixed-sown forage. The rate of increase in the water use efficiency (WUE) of mixed-sown forage was relatively high during 2018–2020. This was mainly due to the improvement in agricultural mechanization levels and the advancement of irrigation techniques, which allowed for more precise control of irrigation water, thereby improving the WUE of mixed-sown forage. In conclusion, the results of this study, combined with the best planting practices provided in this work, including regional, climatic, and temporal factors, will serve as a reference for policy development and agricultural practices, the rational regulation of sowing rate, optimization of soil acidity and alkalinity, and the integration of policy guidance and agricultural technological improvements as important approaches for enhancing the productivity and resource use efficiency of mixed sowing forage. Future plans include conducting multiple trials in different locations and settings, and we will also conduct long-term validation trials to evaluate the applicability and stability of the meta-analysis results. In addition, we note that regional characteristics (e.g., location, rainfall, temperature, altitude, and soil pH) have a significant impact on forage production in this study (Figure 9). Systematic differences in environmental conditions across regions may influence the adaptability and performance of mixed-sowing systems by affecting forage growth conditions and resource use efficiency. Also, while management factors (like the forage mixture and sowing rate) somewhat affect forage yield, the leading role of regional characteristics suggests that mixed-sowing system optimization should be based on specific ecological conditions. Therefore, integrated analysis of regional and management factors can boost forage production efficiency in complex ecosystems.

## 4. Materials and Methods

### 4.1. Data Sources

**Figure 10:**
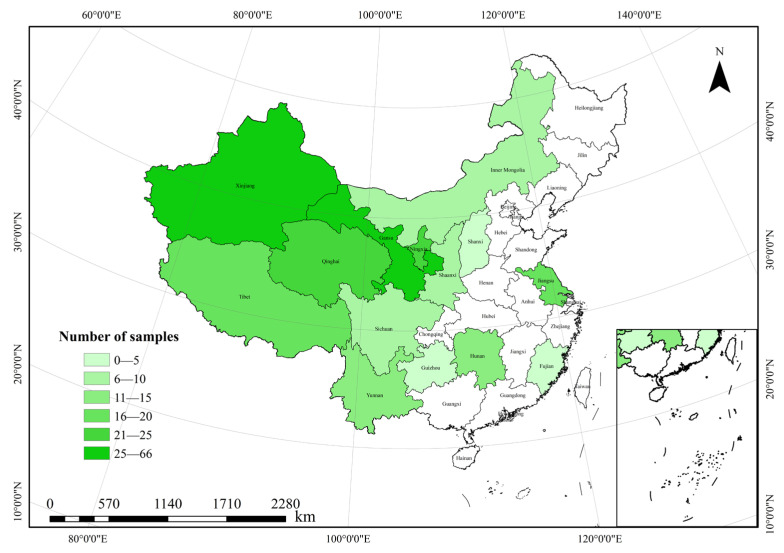
Distribution of regional sample size in this study.

Through employing the Chinese knowledge network (http://www.cnki.net/) and Web of Science (http://apps.webofknowledge.com/) to retrieve databases in both English and Chinese, the Chinese knowledge network: URL: http://apps.webofknowledge.com/ (accessed on 1 March 2023), Web of Science: URL: http://apps.webofknowledge.com/ (accessed on 1 March 2023). experimental studies on the effects of mixed sowing mode on forage yield and water use efficiency published at home and abroad before March 2023 were comprehensively collected. The Chinese search terms included “forage”, “mixed sowing”, “yield”, and “water use efficiency”; the English search terms include “forage”, “mixed sowing”, “yield”, and “water use efficiency”. The search was limited to the title, abstract, and keywords of the paper. In order to reduce the bias caused by literature collection, the analysis samples of this study were selected based on the following criteria: (1) the experiment was located in China and conducted in the field; (2) controls for both the mixture and the monoculture were included; (3) the forage yield and/or water use efficiency for the treatments were listed in the paper, and their mean and standard deviation were directly given, or they could be calculated according to the existing data; (4) the year the experiment was conducted, the type, the proportions of the different species, the method of sowing, and the sowing rate were reported; (5) the rainfall, temperature, elevation, and soil pH for the site were available. After screening using the above criteria, a total of 148 research papers were obtained, including 237 sets of yield data and 78 sets of water use efficiency data. Figure 10 is distribution of regional sample size in this study.

### 4.2. Data Classification

The 148 selected studies primarily involved experimental sites across 14 provinces or autonomous regions in China, including Jiangsu, Fujian, Shanxi, Inner Mongolia, Hunan, Shaanxi, Qinghai, Ningxia, Gansu, Xinjiang, Yunnan, Guizhou, Sichuan, and Tibet. To better interpret the effects of mixed sowing on forage yield and water use efficiency, the selected indicators were classified according to the following 15 factors: provinces/AR, annual average precipitation (mm), annual average temp (°C), altitude (m), composition of mix, no. of species, mixture, legume–grass ratio, sowing method, forage, forage type, sowing rate (kg·ha^−1^), experimental year, soil pH, and total sowing rate (kg·ha^−1^) (Table 2 and Table 3). In addition, the average yield and water use efficiency of monoculture and mixed sowing of forage crops in each province are shown in Table 4 and Table 5. The average altitude, average annual precipitation, average annual temperature, soil pH value, and total seeding rate of each province are shown in Table 6.

### 4.3. Data Analysis

#### 4.3.1. Calculation of Effect Size

Effect size can be used to measure the impact of a specific practice. In this study, the natural logarithm of the response ratio (*R*) was calculated as the effect size to represent the impact of mixed sowing [63]. The effect size *lnR* is expressed as follows:

(1) $$\ln R=\frac{X_{T}}{X_{C}}$$

where *R* is the response ratio, *X_T_* is the forage yield under mixed sowing (kg·ha^−1^), and *X c* is the forage yield under monoculture (kg·ha^−1^).

(2) $$\mathrm{WUE}=\frac{Y}{\mathrm{ET}}$$

where *Y* is the total forage yield (kg/hm^2^); *ET* is the total water consumption (mm). The value of yield is the sum of multiple mowing. In this meta-analysis, weighted analysis was applied. Each study was assigned a specific weight (w, the reciprocal of variance v) to compensate for differences in precision among studies. The weighted effect size (*InR*) and its confidence interval (CI) were calculated based on *InR*, as follows:

(3) $$v=\frac{S_{T}^{2}}{n_{T}X_{T}^{2}}\frac{S_{C}^{2}}{n_{c}X_{C}^{2}}$$

where *S_T_* and *S_C_* are the standard deviations of forage yield or WUE under mixed sowing and monoculture, respectively; *n_T_* and *n_C_* are the respective replication numbers.

(4) $$w=\frac{1}{v}\;$$

(5) $$\ln R^{*}=\frac{\sum_{i=1}^{K}w_{i}\ln R_{i}}{\sum_{i=1}^{k}w_{i}}$$

(6) $$S(\ln R^{*})=\sqrt{\frac{1}{\sum_{i=1}^{K}w_{i}}}$$

(7) $$95\%\mathrm{CI}=\ln R^{*}\pm 1.96S\left(\ln R^{*}\right)\;$$

where K is the number of samples, *w_i_* is the weight of the i-th sample, *lnR_i_* is the effect size of the i-th sample, S is the standard deviation, and R* is the weighted effect size.

To provide a more intuitive understanding of the yield and quality improvement effect of mixed sowing, all results are reported as percentage changes (*Z*) relative to monoculture.

(8) Z= [exp(lnR)−1] ×100%

If the 95% CI does not overlap zero, the mean percentage change is considered significantly different from zero. A positive percentage change indicates an increase in yield or quality under mixed sowing compared to monoculture; otherwise, it indicates a decrease.

#### 4.3.2. Heterogeneity Test

To analyze whether the results of different studies exhibit statistical differences, the Q-statistic was used to assess heterogeneity. When the variance among studies is zero, a fixed-effects model is selected; otherwise, a random-effects model is applied. The calculation formula is as follows:

(9) $$Q=\sum_{i=1}^{K}{w_{i}\left(\ln R_{i}\right)}^{2}-\frac{\sum_{i=1}^{K}{w_{i}\left(\ln R_{i}\right)}^{2}}{\sum_{i=1}^{K}w_{i}}$$

When the *P₀* value (the significance test *p*-value of the *Q*-statistic) < 0.05, a random-effects model is selected; when *Pa* ≥ 0.05, a fixed-effects model is selected

#### 4.3.3. Model Verification

In this study, the publication bias of the model was tested by the fail-safe coefficient. If the loss safety factor is greater than 5k + 10, where k represents the number of studies included in the analysis, it indicates that the results are not affected by publication bias, and the results are highly reliable [64].

### 4.4. Data Processing

Data analysis was performed using R software (v.4.10), and figures were generated using Origin 2021. A significance level of *p* < 0.05 was applied; the random forest analysis is implemented using the “randomForest” package in R.

## 5. Conclusions

(1) Compared with monoculture, mixed sowing significantly increased forage yield and water use efficiency (WUE) by an average of 58.3% and 32.0%, respectively.
(2) In regions such as Shaanxi and Fujian, with an average annual precipitation of 200–600 mm, an average annual temperature of 10–15 °C, and altitudes below 2000 m, the following conditions were conducive to improving the yield effect of mixed sowing: alfalfa + *Bromus inermis*, a two-forage allocation, a mixture with Leguminosae and Gramineae with a species ratio of Leguminosae to Gramineae of 1:1, mixed sowing in the same rows, monoculture of Gramineae such as *Agropyron cristatum*, soil pH of 4–5, and a total sowing rate of 50–100 kg·ha^−1^.
(3) In regions such as Gansu, with an average annual precipitation of 400–600 mm, an annual average temperature of 5–10 °C, and altitudes of 1000–2000 m, the following conditions were conducive to improving WUE: oats + peas, a two-forage allocation, a mixture of Leguminosae and Gramineae with a species ratio of Leguminosae to Gramineae of 1:1, mixed sowing in different rows, monoculture of Gramineae such as *Bromus inermis*, sowing rate of 40–50 kg·ha^−1^, soil pH of 8–9, and a total sowing rate of <50 kg·ha^−1^.

In summary, mixed sowing can significantly enhance forage yield and WUE. Its advantages are particularly evident in areas with an average annual precipitation of 400–600 mm and altitudes of 1000–2000 m, while employing a mixture of Leguminosae and Gramineae with a Leguminosae-to-Gramineae ratio of 1:1.

## Figures and Tables

**Table 1:** Comprehensive effects of mixed sowing on forage yield and water use efficiency.

Index	Model	Increase Rate (%)	95% CI (%)		Effect Size Test		Heterogeneity Test		Safety Factor Against Failure	5k + 10
			LL	UL	*Z*	*P*	*Q*	*P_Q_*		
Yield	REM	58.3	45.5	72.3	10.63	0.000	45,412	0.000	794,737	1090
Water use efficiency	REM	32.0	19.2	46.1	5.33	0.000	42,313	0.000	4626	200

**Table 2:** Classification of yield data.

Primary Factors	Secondary Factors				Grouping			
		1	2	3	4	5	6	7
Regional Factors	Provinces/AR	Xinjiang	Gansu	Ningxia	Jiangsu	Qinghai	Tibet	Others
	Annual average precipitation (mm)	<200	200–400	400–600	600–800	800–1000	1000–2000	>1200
	Annual average temp (°C)	<0	0–5	5–10	10–15	15–20	−	−
	Altitude (m)	<1000	1000–2000	2000–3000	3000–4000	>4000	−	−
Mixed Sowing Factors	Composition of mix	H + O	P + O	A + BI	R + P	Others	−	−
	No. of species	2	3	4	−	−	−	−
	Mixture	Legume/Grass	Grass/Grass	−	−	−	−	−
	Legume–Grass	1:1	1:2	1:3	0:1	−	−	−
	Sowing method	Different rows	Same rows	−	−	−	−	−
Monoculture Factors	Forage	AC	BI	Pea	Alfalfa	R	Oat	Others
	Forage Type	Legume	Grass	−	−	−	−	−
	Sowing rate (kg·ha^−1^)	<40	40–50	>50	−	−	−	−
Other Factors	Experimental Year	<2006	2006–2008	2009–2011	2012–2014	2015–2017	2018–2020	>2020
	Soil pH	4.0–5.0	5.0–6.0	6.0–7.0	7.0–8.0	8.0–9.0	−	−
	Total sowing rate (kg·ha^−1^)	<50	50–100	100–150	>150	−	−	−

Others: Yunnan, Hunan, Sichuan, Inner Mongolia, Shaanxi, Fujian, Shanxi, and Guizhou; AR: autonomous region; AC: *Agropyron cristatum*; BI: *Bromus inermis*; R: ryegrass; H + O: hairy vetch + oats; P + O: peas + oats; A + BI: alfalfa + *Bromus inermis*; R + P: ryegrass + peas.

**Table 3:** Classification of water use efficiency data.

Primary Factors	Secondary Factors			Grouping		
		1	2	3	4	5
Regional Factors	Provinces/AR	Gansu	Qinghai	−	−	−
	Annual average precipitation (mm)	<200	200–400	400–600	−	−
	Annual average temp (°C)	<0	0–5	−	−	−
	Altitude (m)	1000–2000	2000–3000	−	−	−
Mixed Sowing Factors	Composition of mix	P + O	S + BI + R	BI + S	A + BI	Others
	No. of species	2	3	−	−	−
	Mixture	MLAG	MGAG	−	−	−
	Legume–Grass	1:1	1:2	0:1	−	−
	Sowing method	Different rows	Same rows	−	−	−
Monoculture Factors	Forage	Silage Corn	Oat	Alfalfa	BI	Sainfoin
	Forage Type	Leguminosae	Gramineae	−	−	−
	Sowing rate (kg·ha^−1^)	<40	40–50	>50	−	−
Other Factors	Experimental Year	2015–2017	2018–2020	−	−	−
	Soil pH	6.0–7.0	7.0–8.0	8.0–9.0	−	−
	Total sowing rate (kg·ha^−1^)	<50	50–100	100–150	>150	−

P + O: peas + oats; S + BI + R: sainfoin + *Bromus inermis* + ryegrass; BI + S: *Bromus inermis* + sainfoin; A + BI: alfalfa + *Bromus inermis*.

**Table 4:** The average of yield by province.

Provinces/AR	Ningxia		Tibet		Qinghai		Jiangsu		Guizhou		Xinjiang		Hunan	
Average yield (×10^3^ kg·ha^−1^)	Mixed Sowing	Mono- culture	Mixed Sowing	Mono- culture	Mixed Sowing	Mono- culture	Mixed Sowing	Mono- culture	Mixed Sowing	Mono- culture	Mixed Sowing	Mono- culture	Mixed Sowing	Mono- culture
	14.3	12.2	7.6	7.0	17.4	15.2	14.8	11.7	14.7	10.9	12.8	7.4	4.3	2.8
Provinces/AR	Shanxi		Inner Mongolia		Shaanxi		Sichuan		Fujian		Yunnan		Gansu	
Average yield (×10^3^ kg·ha^−1^)	Mixed Sowing	Mono- culture	Mixed Sowing	Mono- culture	Mixed Sowing	Mono- culture	Mixed Sowing	Mono- culture	Mixed Sowing	Mono- culture	Mixed Sowing	Mono- culture	Mixed Sowing	Mono- culture
	10.5	6.4	15.9	8.2	2.9	1.9	16.7	11.2	4.9	1.7	10.1	8.2	10.7	8.8

**Table 5:** The average of water use efficiency by province.

Provinces/AR	Gansu		Ningxia	
Average WUE (kg·m^−3^)	Mixed Sowing	Monoculture	Mixed Sowing	Monoculture
	2.7	2.2	2.6	2.7

**Table 6:** Average values of altitude, annual average precipitation, annual average temp, soil pH, and total sowing rate by province.

Provinces/AR	Ningxia	Tibet	Qinghai	Jiangsu	Guizhou	Xinjiang	Hunan
Altitude (m)	1454.1	4148.0	4112.6	5.0	1468.0	592.0	44.9
Annual average precipitation (mm)	312.8	476.8	598.3	1030.0	1121.4	208.1	1422.4
Annual average temp (°C)	8.4	6.3	−4.6	15.0	12.6	7.9	17.0
Average Soil pH	8.4	7.9	6.9	7.3	5.8	8.6	4.7
Total sowing rate (kg·ha^−1^)	105.2	95.2	182.4	11	2.5	29.8	64.3
Provinces/AR	Shanxi	Inner Mongolia	Shaanxi	Sichuan	Fujian	Yunnan	Gansu
Altitude (m)	1016.1	1800.0	454.8	1118.1	314	187.7	1807.3
Annual average precipitation (mm)	390.0	375	635.1	250.4	1600.5	650.0	282.6
Annual average temp (°C)	6.4	6.2	12.9	11.8	19.2	5.8	7.9
Soil pH	6.8	8.5	8.4	6.1	4.8	6.1	7.5
Total sowing rate (kg·ha^−1^)	28.5	105	12.5	161.1	27.9	187.7	65.0

## Data Availability

All data supporting this study are included in the article.
